# Editorial: Surveying Life One Molecule at a Time: Single Molecule Methods Dig Deeper Into Contemporary Biology

**DOI:** 10.3389/fmolb.2022.967434

**Published:** 2022-07-08

**Authors:** Seong Ho Kim, Sujay Ray, Isaac T. S. Li

**Affiliations:** ^1^ Department of Chemistry, University of British Columbia, Kelowna, BC, Canada; ^2^ Department of Chemistry, University of Michigan, Ann Arbor, MI, United States

**Keywords:** single molecule FRET (smFRET), DNA nanoarchitecture, DNA helicase, single-molecule techniques, molecular tension probe, DNA secondary structures

## Introduction

*“There’s Plenty of Room at the Bottom*,*”* perhaps this invitation from Richard Feynman back in 1959 intrigued a generation of scientists to investigate small molecules using physical techniques. Physicists became interested in understanding how complex biological systems work from their basic building blocks, individual biomolecules. How they fold, move, interact, and how they enable the complex life forms on Earth. They brought in tools from physics, such as microscopes, lasers, photonic detectors, and numerous numerical methods. A new area of biology emerged, known as the single-molecule biophysics, where scientists can now see and manipulate individual biomolecules. The single-molecule toolbox has been applied to study the molecules of life, proteins, lipids, DNA, RNA, and their interactions, literally one molecule at a time, from single cells to complex organisms. These methods have opened new dimensions in our understanding of contemporary biology. In the last decade, it has been possible to do experiments that were fanciful hopes only a couple of decades ago. Investigating one molecule at a time provides clear insights into mechanistic details of the molecular machinery that were previously unattainable due to average blurring in traditional ensemble-averaged bulk studies. Single-molecule methods can provide detailed information about highly dynamic processes, short-lived or transient states, rare molecular events, molecular sub-populations, and non-uniform kinetics in individual molecules. Kinetics is also coming into focus as scientists begin to understand how biological processes are kinetically controlled, as they are more energy-efficient than overcoming considerable thermodynamic energy barriers of stable conformational changes. With advancements in bio-nanotechnology, understanding and manipulating such kinetically controlled systems have become more critical. Single-molecule methods are now widely adopted as an integral approach to contemporary biology.

## Dynamics of DNA Nanostructures

Single-molecule FRET (smFRET) is a powerful method to observe large conformational dynamics of DNA nanostructures on the order of 3–10 nm. Bandyopadhyay and Mishra, in their comprehensive review, described how smFRET was applied to examine the secondary structures of DNA, such as hairpin, Holliday Junction (HJ), G-quadruplex (GQ), and i-motif. Just as a few examples, smFRET helped reveal the folding kinetics and conformational fluctuations of DNA hairpins. Similarly, it enabled studies of HJ dynamics, branch migration, and binding to branch migration enzymes such as the resolvase at the single-molecule level. Additionally, smFRET has helped reveal the multiple conformational states of GQs, their interconversion kinetics and interaction with helicases. Moving to larger DNA nanostructures, Pal, in their mini-review, described the application using smFRET to study the structural dynamics of DNA-based biosensors, drug delivery vehicles, nano-tweezers, walkers, and other bioassays. The large DNA nanostructures described here go beyond a single secondary structure and combine structural motifs and DNA origami structures.

## Dynamics of Helicases

Helicases are motor proteins that translocate on single-stranded DNA to unwind double-stranded DNA during DNA replication. The comprehensive review from Spinks et al. dived deep into the single-molecule insight into the dynamics of replicative helicases. The dynamics of helicases have been studied by a broad range of single-molecule methods, including optical traps, magnetic tweezers, smFRET, single-molecule fluorescence recovery after photobleaching (FRAP), and single-molecule fluorescence loss in photobleaching (FLIP). These single-molecule investigations revealed the intermediate states during unwinding and their dynamic interactions within the replisome–processes that ensemble methods cannot uncover. The review provided an overview of model replisome systems and their helicases from phages, prokaryotic, and eukaryotic cells, and how various single-molecule methods provided novel insights into helicase mechanism, including the active translocation and passive unwinding of DNA, the coordination of their subunits, the processivity and step sizes, as well as their interactions with the replisome.

## DNA Probes at Cellular Interface

One of the applications of DNA nanotechnology in a biological system is the molecular tension probes. The research report from Kim et al. described how cell spreading on anisotropic nanopatterns depends on the molecular tension. The authors used the molecular tension probe on the anisotropic nanopatterns (800 nm ridge × 800 nm groove × 600 nm height) to elucidate how cells react to topographical cues and generate forces. Their experiment compared cellular morphology and distribution of adhesion strength. The DNA-based molecular tension probe labelled with ligand and fluorophore visualizes the forces generated by the receptor through DNA duplex rupture. The probes with different rupture thresholds were applied to two cancer cell lines. The highly invasive cancer cells exhibit significant morphological change and increased force generation, while the non-invasive cells showed fewer alterations in morphological and mechanical properties. Their findings revealed that topographical cues could affect the force applied by integrin at the subcellular level by different types of cell lines.

## New Single-Molecule Methods

New single-molecule methods open opportunities to investigate new biological problems. In their research paper, Dey et al. described a new technique called Q-SLIP (Quenching-induced Step Length Increase in Photobleaching), which combines single-molecule photobleaching and fluorescence quenching to measure the solvent accessibility of proteins and their conformational states. The authors used tryptophan in solution as quenchers for rhodamine-B labelled peptides to study its interaction with a lipid membrane. When the residue labelled by the fluorophore becomes solvent-exposed, its lifetime increases due to exposure to quenchers, and vice versa when the residue becomes embedded within the bilayer. This was observed and quantified in single-molecule photobleaching experiments, providing insight into how the peptide is oriented and embedded in the lipid bilayer.

## Summary and Future Directions

As advanced cutting-edge methods are being developed, single-molecule techniques will continue to make breakthroughs in molecular and cellular biology and decipher even more complex biological processes. However, it is even more fascinating to see the adoption of single-molecule techniques for highly sensitive clinical diagnostics and therapeutics. Single-molecule detection of individual biomarkers can improve disease diagnosis and treatment. The development of single-molecule biosensors opens up opportunities to monitor disease by uncovering hidden properties of individual molecules even at ultralow levels of biomarkers. We are on the verge of a new era of sensitive “at-home” testing devices and personalized medicine involving sophisticated single-molecule techniques.

